# Early Lean Tissue Loss and Nutritional Challenges Following Sleeve Gastrectomy in a Kidney Transplant Recipient: A Case Report

**DOI:** 10.3390/jcm15186940

**Published:** 2026-09-08

**Authors:** Nida Yıldız, Halil Coşkun, Serpil Görçin, Nazlı Batar, Şule Arslan, Halit Tanju Besler

**Affiliations:** 1 Department of Nutrition and Dietetics, Faculty of Health Sciences, Istanbul Aydin University, Istanbul 34295, Turkey; sulearslan2@aydin.edu.tr (Ş.A.); halittanjubesler@aydin.edu.tr (H.T.B.); 2 BariatrikLab Obesity and Metabolic Surgery Center, Istanbul 34093, Turkey; halilcoskun@hotmail.com; 3 Department of Nephrology, Memorial Şişli Hospital, Istanbul 34385, Turkey; serpil.gorcin@memorial.com.tr; 4 Department of Nutrition and Dietetics, Faculty of Health Sciences, Istanbul Kultur University, Istanbul 34158, Turkey; n.batar@iku.edu.tr

**Keywords:** kidney transplantation, sleeve gastrectomy, body composition, lean body mass, bariatric surgery, case report

## Abstract

**Background/Objectives**: Obesity after kidney transplantation impairs graft function, but the time course of lean tissue loss after sleeve gastrectomy in this population is poorly described. **Methods**: A 46-year-old woman underwent sleeve gastrectomy five years after kidney transplantation and was followed for 33 months with bioelectrical impedance analysis (BIA) and three 24-h dietary recalls per visit. **Results:** Weight fell from 103.4 to 69.4 kg by month 6, with mild regain to 71.9 kg at month 33. BIA-derived fat-free mass, BIA-predicted total muscle mass and BIA-derived skeletal muscle mass reached their lowest recorded values at month 3, each 21.3% below baseline, and were essentially unchanged at month 6; fat-free mass loss corresponded to 34.1% of the weight lost by month 6, above the thresholds proposed for excessive loss in a bariatric cohort which are research rather than clinical definitions. This early loss coincided with three concurrent exposures: energy restriction (521 and 466 kcal/day at months 1 and 3), protein intake below target during a period of aversion to protein-rich foods (33.2 g/day against a 60 g/day target), and absence of structured physical activity; anaemia was present throughout and may have contributed to both. Graft function was clinically stable but not unchanged, with estimated glomerular filtration rate 25% below baseline at month 33 and persistent proteinuria; biopsy was not performed. **Conclusions**: Early postoperative nutritional vulnerability was temporally associated with substantial lean tissue loss. The estimates are BIA-derived and hydration-dependent, muscle function was not assessed, and the concurrent exposures cannot be separated in a single case, so no causal relationship can be inferred.

## 1. Introduction

Obesity is a significant clinical issue with an increasingly high prevalence among kidney transplant candidates and recipients [1,2]. Following kidney transplantation, weight gain may occur due to chronic corticosteroid use, lifestyle changes, and metabolic factors [3]. Obesity in kidney transplant recipients is associated with adverse outcomes such as post-transplant diabetes, delayed graft function, and surgical complications [1].

Bariatric surgery is considered an effective option for obesity management both in kidney transplant candidates, in whom it can facilitate access to the waiting list [4], and in patients who have already received a graft [5]. In particular, sleeve gastrectomy is frequently preferred in this patient group because it has a minimal impact on the absorption of immunosuppressive medications and carries a lower risk of postoperative complications [5]. However, gastrointestinal and anatomical changes following bariatric surgery can lead to food intolerances, food aversions, and various nutritional challenges [6,7]. In the general bariatric surgery population, inadequate protein intake has been identified as a modifiable risk factor for fat-free mass loss after sleeve gastrectomy [8]. Existing case series of sleeve gastrectomy in kidney transplant recipients, however, have focused primarily on graft outcomes, body weight, and body mass index, without reporting body composition or the time course of dietary protein intake [9]. Two features of this evidence base are worth distinguishing. First, most of it concerns bariatric surgery performed before transplantation, where the questions asked are access to the waiting list and perioperative safety; in a systematic review of 30 studies of bariatric surgery in end-stage kidney disease or kidney transplantation, 18 addressed patients awaiting a graft and only 14 patients who had already received one, with two studies having included both populations [10]. Second, in the smaller post-transplant literature, the reported outcomes are percentage excess weight loss, comorbidity resolution, creatinine or estimated glomerular filtration rate, proteinuria, complications and mortality [10]. The same pattern holds at population level: in a matched national analysis of 770 kidney transplant recipients, of whom 155 underwent bariatric surgery, graft failure and death were the outcomes reported, and both were favourable after surgery [11]. Body composition and quantified dietary intake do not appear among the outcomes assessed in either source [10,11]. Safety and transplant outcomes have thus received considerable attention in this population [9,10,11], whereas the course of lean tissue and its timing appear to have been examined less often. As a result, data on the magnitude of fat-free mass and skeletal muscle mass loss, its temporal relationship with modifiable factors such as inadequate protein intake, and its long-term trajectory specifically in kidney transplant recipients remain limited. In particular, longitudinal data linking the time course of dietary intake to body composition changes in this population, beyond the expected degree of lean tissue loss described in the general bariatric surgery literature [8], appear to be scarce. Three settings must therefore be kept apart when this literature is read: bariatric surgery in the general population, bariatric surgery performed before kidney transplantation, and bariatric surgery performed after transplantation. Only the third applies directly to the present case, and estimates derived from general bariatric cohorts are used below for orientation rather than as directly applicable reference values. Therefore, this case report presents the long-term nutritional outcomes, body composition changes, and clinical follow-up of a kidney transplant recipient who underwent sleeve gastrectomy, with particular attention to the timing of early fat-free and skeletal muscle mass loss and to the nutritional exposures present over the same interval.

This case report was prepared in accordance with the CARE (CAse REport) guidelines [12], and a completed CARE checklist is provided as a Supplementary File. Three separate requirements were met. Written informed consent for publication of this case report and the accompanying clinical data was obtained from the patient. Ethical approval for the use of the clinical data, together with institutional permission for retrospective publication of the case, was granted by the Ethics Committee of Memorial Şişli Hospital, Istanbul, Turkey (Approval No: 003, Date: 22 September 2025). All care described here was delivered as routine clinical practice; no procedure was performed for research purposes, and the approval was sought after the follow-up period had ended, for publication only.

## 2. Materials and Methods

### 2.1. Timeline

The clinical course of the patient from the initial diagnosis of chronic kidney disease to the most recent follow-up visit is summarised in Table 1. The timeline covers the pre-transplant period, kidney transplantation, the subsequent weight gain leading to bariatric surgery, and the 33-month postoperative follow-up, and indicates the visits at which nutritional assessment, body composition analysis and biochemical evaluation were performed.

### 2.2. Patient Information and Clinical Findings

A 46-year-old female patient presented to the obesity and metabolic surgery centre of a private hospital due to morbid obesity. She was diagnosed with chronic kidney disease of unknown etiology in 2006 and underwent a pre-emptive living-donor kidney transplantation from her spouse in 2017; sleeve gastrectomy was performed in 2022, approximately five years after transplantation. She was an active smoker at the preoperative assessment; because smoking status was not reassessed at any postoperative visit, smoking is reported here only as a baseline risk factor, and no inference is drawn about its contribution to events occurring during follow-up. Physical activity was asked about at every visit as part of the routine dietetic consultation and recorded in the clinical notes; the patient reported no structured physical activity at any assessment. This assessment was qualitative. No validated physical activity questionnaire was administered, and habitual step count, occupational and household activity, sedentary time and any attempt at resistance exercise were not quantified. The descriptor “no structured physical activity” therefore denotes the absence of planned exercise and is not equivalent to documented physical inactivity. Current guidance for this population recommends that physical activity be assessed both subjectively and objectively, since self-reported and device-derived estimates diverge [13]; only the subjective component was available in this patient. Prior to bariatric surgery, her body weight was 103.4 kg, height was 161 cm, and body mass index was 39.9 kg/m^2^. Comorbidities included hypertension, obstructive sleep apnea, knee pain, and insulin resistance.

Body composition parameters were assessed by multifrequency bioelectrical impedance analysis (BIA) with a Tanita MC-780MA analyzer (Tanita Corporation, Tokyo, Japan). The following device outputs were extracted at each visit: fat-free mass, muscle mass, skeletal muscle mass, body fat mass, total body water, intracellular water and extracellular water. Body weight at each visit was the value measured on the analyser at the time of the impedance assessment, and all body composition values were transcribed directly from the device printout for that visit. According to the manufacturer, the muscle mass output represents predicted total muscle mass, includes smooth and cardiac muscle and the water contained within these tissues [14], and is therefore not equivalent to skeletal muscle mass as quantified by dual-energy X-ray absorptiometry or magnetic resonance imaging; skeletal muscle mass is reported by the device as a separate output, alongside an organ muscle output. Fat-free mass as reported by the device corresponds to muscle mass together with bone mineral content. These three outputs are reported separately throughout and are not used interchangeably: fat-free mass, BIA-predicted total muscle mass and BIA-derived skeletal muscle mass each denote a distinct device estimate, and none of them is a direct anatomical measurement. All measurements were obtained on the same device by the same operator. Assessments were performed between 10:51 and 13:53 h; fasting state, time since last fluid intake and bladder status were not systematically standardised across visits, and this is addressed in Section 4.5. Daily energy and macronutrient intakes were estimated from three 24-h dietary recalls (two weekdays and one weekend day), prospectively collected at each follow-up visit as part of routine clinical care. All recalls were conducted in person by the same treating dietitian throughout. Mean daily intake values were calculated from the three recalls for each period and analysed using the Nutrition Information System software (BeBiS, version 9.0; Ebispro for Windows, Stuttgart, Germany) [15]. The same two-weekday and one-weekend-day structure was applied at every visit. Portion sizes were estimated using a Turkish food and meal photograph catalogue of standard measures and quantities [16]. The prescribed oral whey protein supplement was included in the reported energy and macronutrient totals on the days on which the patient reported having taken it, so the values in Table 2 represent combined intake from food and supplements rather than from food alone. The values are estimates of intake actually consumed, as reported by the patient, and are not prescribed or planned intakes. They include all foods and all energy-containing beverages reported, so the very low early postoperative figures are not an artefact of excluding fluids or supplements; intravenous fluids given during hospital admissions are not included. Weight loss outcomes were expressed according to the standardized reporting criteria of the American Society for Metabolic and Bariatric Surgery [17] as the change in body mass index (ΔBMI), percentage total weight loss (%TWL = [preoperative weight − current weight]/preoperative weight × 100) and percentage excess weight loss (%EWL = [preoperative weight − current weight]/[preoperative weight − ideal body weight] × 100). For the weight loss metrics, ideal body weight was taken as the weight corresponding to a body mass index of 25 kg/m^2^, as specified in that document [17], giving 64.8 kg for this patient and an excess weight of 38.6 kg. A different estimate of ideal body weight, conventional in clinical nutrition, is used for the protein requirement calculations reported in Section 4.3. Estimated glomerular filtration rate was calculated from serum creatinine at each visit using the race-free 2021 CKD-EPI creatinine equation [18], applying the patient’s sex and her age at each sampling date (46 years preoperatively and at months 1, 3 and 6; 49 years at month 33). Since serum creatinine exceeded 0.7 mg/dL at every visit, the applicable form of the equation for women was eGFR = 142 × (serum creatinine/0.7)^−1^·^200^ × 0.9938^age × 1.012, with serum creatinine in mg/dL and age in years.

### 2.3. Perioperative and Postoperative Management

Following multidisciplinary evaluation, the patient underwent laparoscopic sleeve gastrectomy in 2022 and was managed according to the standard postoperative bariatric dietary protocol used at the treating centre. That protocol follows the staged progression set out in current practical guidance [13]. Clear, non-carbonated and sugar-free liquids were begun on the first postoperative day once a leak had been excluded, taken in small and frequent volumes, and sugar-free liquids together with chicken and meat broth were continued on days 2 and 3. From day 4 until days 10–14 the diet consisted of full liquids, including milk, yogurt and whey-based protein supplements at 25–30 g per serving. Pureed and soft protein-rich foods were introduced between days 10–14 and the end of the third week, taken as three small meals with two snacks, with fluids separated from meals. From the fourth postoperative week the diet was advanced according to individual tolerance, with new foods reintroduced singly and a fluid intake of at least 1500–1800 mL/day. A standard laparoscopic sleeve gastrectomy was performed, and the patient was monitored for postoperative surgical complications, including staple line leak, none of which occurred. A whey protein supplement containing 27 g of protein per packet (Barivital; Pharmago Health Products and Foreign Trade Inc., Istanbul, Türkiye) was prescribed as one packet daily from the full-liquid stage onward, to be continued until the protein target could be met from food alone, and was reduced to half a packet according to tolerance. Its renal safety at the prescribed dose was discussed with the patient at the postoperative visits; actual use is described in Section 3.1. Venous thromboembolism prophylaxis consisted of subcutaneous enoxaparin sodium 40 mg (4000 anti-Xa IU) once daily throughout the first 10 postoperative days, continued after discharge until completion of that period, with no extended prophylaxis thereafter; mechanical prophylaxis with graduated compression stockings was applied for at least the first 4 postoperative days. Doppler ultrasonography was performed in the radiology department of the treating hospital as part of routine clinical care; the specific device model was not recorded in the patient’s file and could not be retrieved retrospectively. Immunosuppressive therapy with tacrolimus, mycophenolate mofetil and prednisolone was continued throughout, together with bisoprolol for hypertension. Vitamin and mineral supplementation was prescribed as monthly intramuscular vitamin B12, monthly vitamin D, daily folic acid, a multivitamin preparation and intravenous iron.

Generative artificial intelligence (AI) tools—ChatGPT (GPT-5.6, OpenAI, San Francisco, CA, USA), DeepL Write (DeepL SE, Cologne, Germany), and Claude (Claude Opus 5, Anthropic, San Francisco, CA, USA)—were used solely to improve the readability, language and structural formatting of the manuscript. They were not used for data collection, data analysis, or interpretation of the findings. The authors reviewed and edited all output and take full responsibility for the content of this publication.

## 3. Results

### 3.1. Postoperative Course and Treatment Adherence

Body composition parameters over the 33-month follow-up are presented in Table 3. Adherence to the prescribed regimens was incomplete. The patient did not use the whey protein supplement regularly because of concern that it might impair kidney function. Serum tacrolimus trough levels fluctuated during follow-up (Table 4). The records available for this retrospective report did not consistently document the tacrolimus dose at each assessment, the dosing schedule, the interval between the preceding dose and blood sampling, or the target trough range applied by the treating transplant team, and dose changes made between visits could not be reconstructed; mycophenolate mofetil and prednisolone doses were likewise not consistently recorded. The specific contributions of dose adjustment, postoperative absorption changes and medication adherence to the observed fluctuation therefore could not be determined. Immunosuppressive therapy was managed by the transplant unit rather than by the reporting centre, and the corresponding prescribing records were not part of the documentation available for this report; the tacrolimus concentrations are therefore reported as measured values without the pharmacokinetic context required to interpret them. Records indicated that the prescribed vitamin and mineral regimen was followed for only approximately one month. Intravenous ferric carboxymaltose 500 mg was administered at postoperative month 2.

### 3.2. Nutritional Assessment and Dietary Intake

During the first 3 postoperative months, the patient was unable to meet the recommended daily protein intake, with protein intake declining further from postoperative month 1 to its lowest point at postoperative month 3 (Table 2); intake began to increase from postoperative month 6 onward, reaching 59 g/day at month 6 and 57.4 g/day at month 33. These values are at or marginally below the 60 g/day target, differing from it by 1.0 and 2.6 g/day respectively, and are best described as approximating the target rather than falling short of it. The patient reported an aversion to protein-rich foods, including meat, chicken, and fish, beginning approximately 1 month after surgery; this aversion, along with accompanying taste and smell sensitivity, appeared to ease by approximately postoperative month 6, coinciding with the rise in measured protein intake at that time point. According to the patient’s self-report, an increase in the consumption of sweets, chocolate, and cakes began approximately one year after surgery and continued during the period of weight regain at long-term follow-up, consistent with the higher carbohydrate and fat intake recorded at the postoperative month 33 dietary recall (Table 2). Energy intake was 2922 kcal/day preoperatively, 521 kcal/day at month 1, 466 kcal/day at month 3, 780 kcal/day at month 6 and 1541 kcal/day at month 33 (Table 2); intake therefore declined between months 1 and 3 before increasing.

### 3.3. Body Composition and Biochemical Outcomes

**Figure 1:**
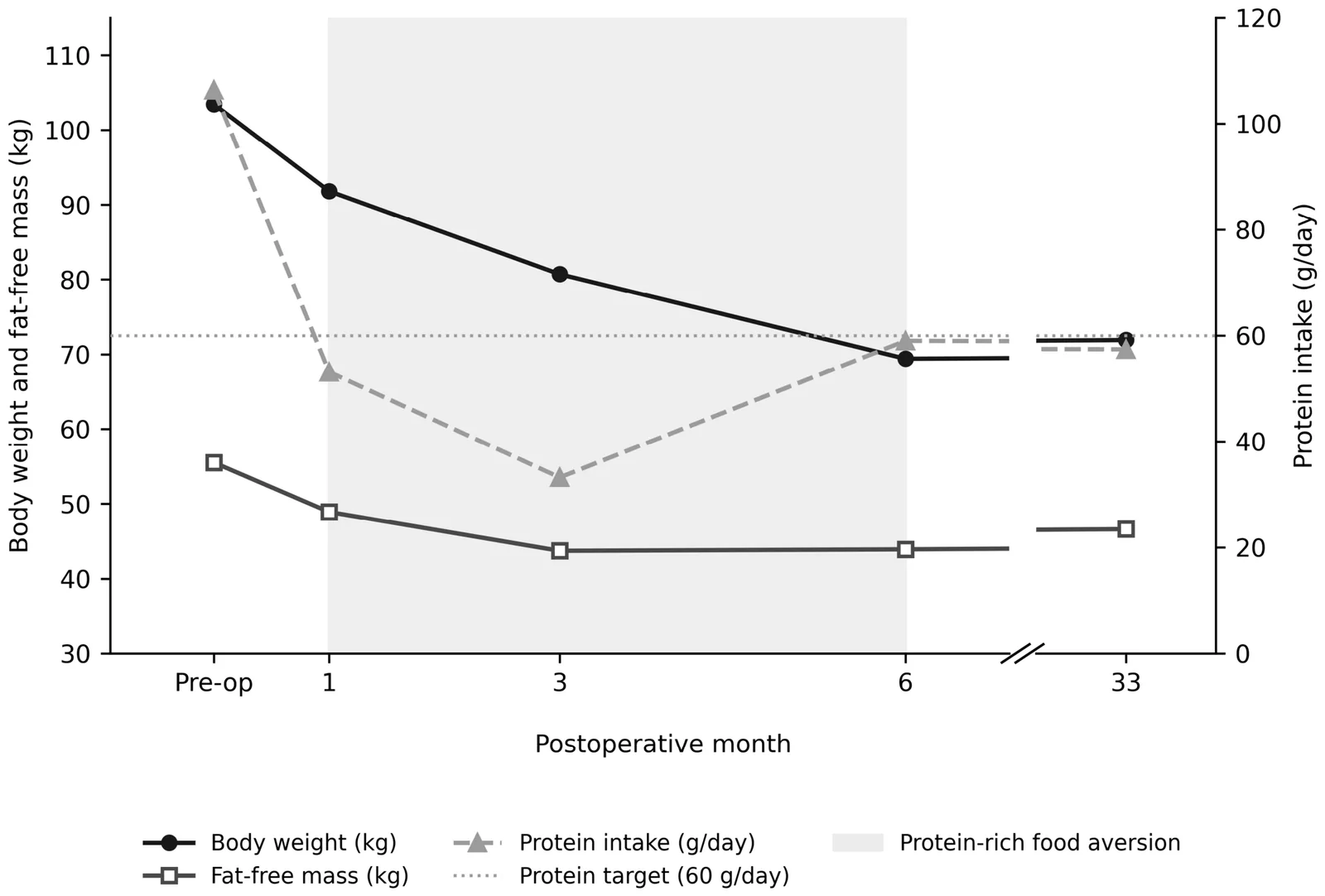
Time course of body weight, fat-free mass and dietary protein intake before and after sleeve gastrectomy. Body weight and fat-free mass are plotted on the left axis and mean daily protein intake on the right axis. The dotted line indicates the 60 g/day protein target used for counselling; the shaded band spans the period of self-reported aversion to protein-rich foods, from month 1, when it developed, to month 6, by which time it had eased. Note the break in the horizontal axis between months 6 and 33.

The early postoperative period was uneventful. At postoperative month 3, deep vein thrombosis of the left lower limb was diagnosed by Doppler ultrasonography. The clinical records document the site and the diagnostic modality but do not specify the presenting symptoms or the venous segment involved or whether the thrombosis was proximal or distal, and no thrombophilia workup is recorded. Therapeutic anticoagulation with subcutaneous enoxaparin sodium 60 mg (6000 anti-Xa IU) once daily, at a dose adjusted for the patient’s renal function, was started and continued for six months, together with micronised purified flavonoid fraction (diosmin and hesperidin) 500 mg twice daily; graduated compression stockings were worn for one year. Symptoms resolved within approximately one week of starting treatment. The thrombotic event, the associated limb symptoms and the accompanying acute inflammatory response coincided with the month 3 assessment, at which dietary intake, body composition and biochemistry were all recorded; the implications for the interpretation of those data are considered in Section 4.3 and Section 4.5. Body weight decreased to 69.4 kg and body mass index to 26.8 kg/m^2^ by postoperative month 6. The decrease in fat mass was accompanied by a loss of fat-free mass, muscle mass and skeletal muscle mass (Table 3). All three reached their lowest recorded values at postoperative month 3 (43.7, 41.5 and 24.7 kg, respectively), with no further decline between months 3 and 6 (43.9, 41.7 and 24.9 kg). The time course of body weight, fat-free mass and dietary protein intake across the five assessments is shown in Figure 1. The patient did not regularly continue dietitian follow-ups after the 6-month visit; she returned at 33 months with a body weight of 71.9 kg and body mass index of 27.7 kg/m^2^. No clinical signs suggestive of acute rejection were observed during long-term follow-up. Serum creatinine rose from 1.09 mg/dL preoperatively to a peak of 1.55 mg/dL at month 3 and was 1.37 mg/dL at month 33; the corresponding estimated glomerular filtration rates were 63, 42 and 47 mL/min/1.73 m^2^ (Table 4). Proteinuria was present before surgery and at every postoperative assessment, with urine protein-to-creatinine ratios of 356 mg/g preoperatively and 395, 325, 288 and 423 mg/g at months 1, 3, 6 and 33 respectively, all above the laboratory upper reference limit. Panel reactive antibody and donor-specific antibody testing, both performed at postoperative month 4, that is, approximately one month after the transfusion described above, were negative. Neither test was repeated over the remaining 29 months of follow-up. Graft biopsy was not performed at any time point. Serum 25-hydroxyvitamin D was 12 ng/mL at postoperative month 3, a deficient value. Both vitamin D and folate formed part of the routine postoperative laboratory panel and were requested at the subsequent visits, but the patient did not attend for sampling, so no values were obtained for vitamin D at months 6 and 33 or for folate at month 33. The NA entries in Table 4 therefore denote tests that were ordered but never performed rather than results that were unavailable for other reasons. Haemoglobin ranged from 9.4 g/dL at month 3 to 11.3 g/dL at month 1 and was 11.1 g/dL at month 33, falling below the laboratory reference range at months 3 and 6 and remaining in the lower part of that range at the other assessments. Intravenous ferric carboxymaltose 500 mg was administered at postoperative month 2, and two units of red blood cells were transfused at month 3 for symptomatic anaemia. Serum ferritin was 19 ng/mL preoperatively, rose to 262 ng/mL at month 3 and was 26 ng/mL at month 33. C-reactive protein followed the same pattern, with the highest value (43.6 mg/L) recorded at month 3 and the lowest (1.7 mg/L) at month 33 (Table 4).

## 4. Discussion

### 4.1. Weight Loss and Body Composition Outcomes

Bariatric surgery is considered an effective approach for managing obesity-related complications in kidney transplant recipients [5]. In the present case, substantial weight loss was achieved following sleeve gastrectomy with no clinical signs of acute rejection. Weight loss itself was of the magnitude expected after sleeve gastrectomy: %TWL was 32.9% and %EWL 88.1% at postoperative month 6, and 30.5% and 81.6% respectively at 33 months (Table 3). The value at month 6 lies above the 13–85% range reported for bariatric surgery performed after kidney transplantation in the systematic review cited above, although that range aggregates values recorded at differing follow-up intervals across the 14 post-transplant studies rather than at a single time point, so the comparison is approximate; in the authors’ own series, three of four patients operated on after transplantation achieved at least 100% excess weight loss [10]. The concern raised by this case therefore relates to the composition of the weight lost rather than to its amount. Renal function, however, was not unchanged. Estimated glomerular filtration rate fell from 63 mL/min/1.73 m^2^ preoperatively to 42 mL/min/1.73 m^2^ at month 3, recovered to 60 mL/min/1.73 m^2^ at month 6, and was 47 mL/min/1.73 m^2^ at 33 months, approximately 25% below the preoperative value (Table 4). Proteinuria was present before surgery (356 mg/g) and remained within a similar range at every postoperative assessment (288–423 mg/g), so it was neither a new finding nor one that changed substantially over follow-up; the decline in estimated filtration therefore occurred against a background of pre-existing proteinuria. Panel reactive antibody and donor-specific antibody testing at month 4 were both negative. Graft biopsy was not performed, so subclinical rejection could not be excluded.

This limitation interacts directly with the body composition findings reported here. Creatinine generation is proportional to muscle mass, and plasma creatinine after bariatric surgery may be lower than expected because of the reduction in muscle mass accompanying weight loss. The substantial reduction in muscle mass documented here may therefore have reduced creatinine generation and may in turn have caused creatinine-based estimates of glomerular filtration to overstate true filtration. Cystatin C, measured glomerular filtration rate and other muscle-independent assessments were not available in this patient, so this remains a theoretical consideration rather than an effect demonstrated in the present data, and neither its presence nor its magnitude can be quantified here. Only 3 of 14 studies of bariatric surgery after kidney transplantation included in that systematic review reported the evolution of proteinuria [10]; the serial ratios reported here therefore add a measure that is seldom available in this setting. We describe the graft course as clinically stable, and not as evidence of preserved graft function.

A further observation bears on this question. Serum urea rose to 62 mg/dL at postoperative month 3, the visit at which protein intake was lowest (33.2 g/day). A diet-driven rise in urea would be expected to accompany higher, not lower, protein intake, so a dietary origin is unlikely. That visit also coincided with the peak creatinine value, with a deep vein thrombosis, with documented but unquantified reduction in fluid intake, and with the highest recorded C-reactive protein concentration (43.6 mg/L). The patient was an active smoker at the preoperative assessment, a recognised risk factor for venous thromboembolism, although her smoking status during follow-up was not documented. A transient haemodynamic component, in the form of volume depletion with reduced graft perfusion against a background of systemic inflammation, is possible. It is not established: acute rejection, calcineurin inhibitor nephrotoxicity, infection and other causes cannot be excluded in the absence of a biopsy, and the available data do not allow these possibilities to be ranked against one another. The fluid-related outputs from the impedance assessment at that visit were nevertheless unremarkable, with a hydration fraction of 72.3% and an ECW/TBW ratio of 0.49, both within the range recorded at the other visits (Section 4.5); the biochemical and impedance data therefore do not point in the same direction. Both the urea and the creatinine values returned toward baseline by month 6, which is compatible with a reversible process rather than with established structural graft injury, although no aetiology can be confirmed without biopsy.

Serum tacrolimus trough concentrations fluctuated during follow-up and were 3.3 ng/mL at month 6 and 4.8 ng/mL at 33 months, both below the trough range of 5.0–6.9 ng/mL reported at cohort level to be associated with better allograft outcomes between two and six years after transplantation [19]. That comparison is descriptive. A trough concentration lying below a range derived from a cohort does not establish inadequate immunosuppression in an individual patient, particularly where the dose, the dosing schedule and the interval between dose and sampling are not documented (Section 3.1), and no such inference is drawn here. Data on tacrolimus pharmacokinetics after sleeve gastrectomy in transplant recipients remain limited and inconsistent [20], and the retrospective design of this report did not allow the relative contributions of dose adjustment, altered absorption and adherence to be determined. Considerable weight loss alters the volume available for drug distribution and may require dose adjustment, and regular monitoring of serum immunosuppressant concentrations has been recommended both in the early postoperative period and until weight stabilises [10]. Immunological monitoring in this patient consisted of serial trough concentrations together with a single donor-specific antibody measurement at month 4, which was negative; protocol biopsy is not performed in the absence of a clinical indication. The immunological course observed here is therefore described rather than interpreted, and does not by itself establish that sleeve gastrectomy is without immunological consequence in transplant recipients.

Weight loss was nonetheless accompanied by clinically relevant reductions in BIA-derived fat-free mass, BIA-predicted total muscle mass and BIA-derived skeletal muscle mass, highlighting the importance of body composition monitoring in addition to weight loss assessment.

### 4.2. Food Intolerance and Protein Aversion

Various nutritional complications may develop due to anatomical and physiological changes following bariatric surgery [21]. Consistent with previous reports, the patient developed self-reported aversion to protein-rich foods, particularly meat, chicken, and fish, beginning approximately 1 month after surgery and accompanied by increased taste and smell sensitivity, with both appearing to ease by approximately postoperative month 6. This time-limited pattern aligns with the lowest protein intake values observed at postoperative months 1 and 3 (Table 2) and with the subsequent partial recovery in intake from month 6 onward. Sensory and appetite changes of this kind are common after bariatric surgery and do not appear to be specific to the procedure performed. In a cross-sectional study of 146 patients assessed a mean of five years after surgery, changes in appetite were reported by 76%, changes in taste by 48.6%, changes in smell by 24% and food aversion by 16.4%, with no difference between sleeve gastrectomy and Roux-en-Y gastric bypass. Among the 20 patients who had undergone sleeve gastrectomy, food aversion was reported by 20% and an increased sensation for sweet taste by 76.5% [22]. Two features of that report bear on the present case. First, sensory changes were more frequent in patients assessed closer to surgery [22], which is consistent with the time-limited course observed here. Second, the shift toward sweet foods reported by this patient from approximately one year after surgery, discussed in Section 4.4 in relation to weight regain, corresponds to the most frequently reported taste change in that cohort rather than representing an isolated change in eating behaviour. Meat is the food group most frequently reported as difficult to ingest after bariatric surgery, and red meat in particular is described as the commonest source of newly acquired taste aversion in the early postoperative months. Altered gut hormone profiles and rapid weight loss are thought to modulate both taste perception and central food reward pathways, so that aversion may be provoked by the smell as well as the taste of meat. Disorders of taste and smell perception are listed among the determinants of meat intolerance, alongside mechanical and digestive factors [6]. The nutritional consequence is twofold: intolerance either reduces protein and micronutrient intake or shifts consumption toward easily tolerated, energy-dense foods [6]. Both patterns were observed in this patient in sequence, the first during the initial three months and the second in the later period discussed in Section 4.4. Graded strategies are recommended for reintroducing meat, including finely minced or slow-cooked preparations, meat served within soups or stews, small portions eaten slowly and thorough chewing, with fish, eggs, legumes and dairy products as alternative protein sources [6]. These strategies were advised at the postoperative visits and the patient agreed to attempt them, but she remained unable to consume the modified preparations during the period of aversion. The timing is what makes this observation relevant here. Bariatric surgery leads to a loss of approximately 8 kg of lean body mass within the first year, a substantial proportion of which occurs in the initial three months and coincides with a period of reduced energy and protein intake; the early postoperative period has therefore been identified as the optimal opportunity for interventions targeting preservation of lean body mass [7]. In this patient the aversion resolved spontaneously by month 6, but the loss of fat-free mass had largely taken place by then.

### 4.3. Nutritional Intake and Loss of Fat-Free Mass

According to the American Society for Metabolic and Bariatric Surgery (ASMBS) guidelines, a daily protein intake of at least 60 g is recommended to maintain lean body mass following bariatric surgery [23]. In kidney transplant recipients undergoing bariatric surgery, higher requirements have been reported (≥1.1 g/kg ideal body weight) [24]; the recommendation is thus expressed per kilogram of ideal rather than actual body weight. This figure was chosen because it is drawn from the only guidance identified that addresses bariatric surgery specifically in patients with chronic kidney disease and kidney transplantation, rather than from the general bariatric or the general transplant literature, and because it applies to stable recipients with preserved or moderately reduced graft function. That description fits this patient: estimated glomerular filtration rate was 63 mL/min/1.73 m^2^ preoperatively and 42–60 mL/min/1.73 m^2^ during follow-up, corresponding to CKD stages G2 to G3b, with stable, non-nephrotic proteinuria throughout, so the target was not reduced on renal grounds. The target was agreed between the dietitian responsible for the patient’s bariatric follow-up and the treating nephrologist. Ideal body weight for this purpose was estimated with the Hamwi formula, defined for women as 100 lb plus 5 lb for each inch above 5 feet, as set out in a commentary on the ideal body weight model [25], giving 53.0 kg for this patient, which differs from the reference weight applied to the weight loss metrics in Section 2.2. On that basis the recommendation corresponds to a target of 58.3 g/day. This is close to the 60 g/day ASMBS threshold, which was the target actually used for counselling in routine clinical care; the two targets were therefore practically equivalent for this patient. Expressed per kilogram of ideal body weight, intake was 2.00 g/kg preoperatively and 1.00, 0.63, 1.11 and 1.08 g/kg at months 1, 3, 6 and 33 respectively; per kilogram of actual body weight, the corresponding values were 1.03, 0.58, 0.41, 0.85 and 0.80 g/kg (Table 2). The two denominators diverge over follow-up because actual body weight fell by up to 34 kg, so part of the apparent recovery in intake per kilogram of actual weight reflects the shrinking denominator rather than increased consumption; the values normalised to ideal body weight are therefore the more appropriate basis for comparison with the recommended target. The patient was unable to meet this target during the first 3 postoperative months, with protein intake declining from 53.1 g at month 1 to its lowest point of 33.2 g at month 3, coinciding with the period of most pronounced food intolerance. From month 6 onward, protein intake improved to 59.0 g/day at month 6 and 57.4 g/day at month 33, corresponding to 1.11 and 1.08 g/kg ideal body weight; intake at month 6 therefore met the transplant-specific target of 1.1 g/kg, while intake at month 33 fell marginally below it. The differences from the target are well under 1 g/day in both directions and are best interpreted as intake having returned to approximately the recommended level rather than as clearly exceeding or falling short of it. An oral protein supplement had been prescribed for this purpose but could not be taken regularly, because of the patient’s concern that it might harm her graft; the deficit during the first three postoperative months, the interval in which the loss occurred, was therefore never corrected. The underlying tension is nevertheless real: postoperative recommendations to increase protein intake and the long-standing concern about renal protein load in transplant recipients pull in opposite directions in the same patient, and this patient’s own belief that supplementation might damage her graft proved to be the decisive determinant of non-adherence.

Inadequate protein intake following sleeve gastrectomy has been identified as a modifiable risk factor for greater fat-free mass loss in a prospective cohort of patients followed for 12 months [8], and inadequate protein intake is among the recognised long-term nutritional problems after bariatric surgery more generally [21]. Fat-free mass reached its lowest recorded value at postoperative month 3 (43.7 kg), as did muscle mass (41.5 kg) and skeletal muscle mass (24.7 kg), each having fallen by 21.3% from the respective baseline value; values at month 6 were essentially unchanged (43.9, 41.7 and 24.9 kg), each differing by 0.2 kg from the corresponding month 3 value. These are impedance-derived estimates, and their interpretation is qualified by the considerations set out in Section 4.5. These figures express loss relative to baseline compartment mass and are therefore not directly comparable with the benchmark most frequently cited in the bariatric literature, which expresses fat-free mass loss as a proportion of total weight lost. The reference cohort described below expresses this quantity as %FFML/WL, calculated as the change in fat-free mass divided by the change in body weight between the preoperative assessment and the follow-up visit, multiplied by 100 [26]. Applying that formula to the present data gives 52.0% at month 3 (11.8 kg of 22.7 kg) and 34.1% at month 6 (11.6 kg of 34.0 kg). Of the two, the month 6 value is numerically the more robust: its denominator is 34.0 kg against 22.7 kg at month 3, so the month 3 ratio moves appreciably with small changes in either term. By way of illustration, a difference of 1 kg in the fat-free mass estimate alters the month 3 ratio by approximately 4.4 percentage points but the month 6 ratio by 2.9, so the month 3 value is the more sensitive to the measurement error discussed in Section 4.5. The month 3 assessment also coincided with the deep vein thrombosis, the documented reduction in fluid intake and the highest recorded C-reactive protein concentration (Section 4.1). That coincidence is unlikely to be neutral for either dataset. An acute thrombotic event with limb symptoms, a systemic inflammatory response, the accompanying reduction in mobility and the initiation of therapeutic anticoagulation could each plausibly have depressed dietary intake around that visit, and acute illness with altered fluid balance is precisely the circumstance in which impedance-derived estimates are least reliable (Section 4.5). Serum magnesium was also below the reference range at that visit (1.3 mg/dL), having been within range at every other assessment. The month 3 values are therefore best read as having been obtained during an acute illness rather than under stable conditions. Both values nevertheless exceed the thresholds described below. In a cohort of 3596 patients undergoing sleeve gastrectomy or Roux-en-Y gastric bypass, in whom body composition was assessed by BIA, mean %FFML/WL was 23.0% at 3 months, fell to 20.9% at 9 months and rose to 24.7% by 36 months; cutoff values of ≥25%, ≥30% and ≥35% have been proposed to define excessive fat-free mass loss [26]. Using the 25% cutoff, 43.3% of that cohort met the definition at 3 months, a prevalence that fell to 28.3% at 12 months and rose to 46.2% at 36 months. Across all follow-up points, prevalences ranged from 12.7% to 25.9% for the 30% cutoff and from 6.9% to 15.8% for the 35% cutoff. The values observed in the present patient exceed all three thresholds at month 3, and the 25% and 30% thresholds at month 6, according to the thresholds proposed by Nuijten et al. [26]. These are research-derived definitions used to describe the distribution of fat-free mass loss within a bariatric cohort; they are not validated clinical diagnostic criteria for pathological muscle wasting, and exceeding them is not equivalent to a diagnosis. Three features of that cohort limit comparability, and none is minor: body composition was measured with a Tanita BC-420MA analyser rather than the MC-780MA used here, so the device-specific predictive equations differ; only 16% of its patients underwent sleeve gastrectomy, the remainder having undergone Roux-en-Y gastric bypass; and the cohort contained no transplant recipients. The month 3 ratio in the present case also rests on a denominator of only 22.7 kg of weight loss, so it moves appreciably with small errors in either term. Notably, sleeve gastrectomy was itself identified as an independent determinant of greater proportional fat-free mass loss in that analysis [26]. A systematic review of 59 studies, in which whole-body dual-energy X-ray absorptiometry, computed tomography or magnetic resonance imaging was an inclusion criterion and bioelectrical impedance was excluded, aggregated data from 2270 individuals; 20 of those studies reported fat-free mass. In that review, the weighted average proportional loss of fat-free mass relative to weight loss at 12 months was 20.8%, a descriptive estimate derived from study-level means rather than a pooled meta-analytic value [27]. Neither dataset was derived from transplant recipients, and immunosuppressive therapy was not reported in either cohort, so applicability to the present patient remains uncertain and the comparison should be read as indicative rather than definitive. The temporal sequence, however, does not align as closely as a causal reading would require. Fat-free mass fell by 6.6 kg during the first postoperative month, when protein intake was 53.1 g/day, and by a further 5.2 kg between months 1 and 3; the majority of the total loss had therefore already occurred before the nadir of protein intake was recorded at month 3 (33.2 g/day). Between months 3 and 6 fat-free mass was essentially unchanged despite intake recovering to 59 g/day, and recovery became apparent only at 33 months, more than two years later. Intake and lean mass thus co-occurred within a partially overlapping window rather than moving in step, and the direction of the relationship cannot be established from a single case. Several factors other than protein intake plausibly contributed, and energy restriction merits at least equal weight. Measured energy intake was 521 kcal/day at month 1, 466 kcal/day at month 3 and 780 kcal/day at month 6. Energy intake of this order is anticipated after bariatric surgery. Current practical guidance defines no specific calorie target and recommends approximately 400–500 kcal/day in the first postoperative days, a gradual increase to below 900 kcal/day over the first six months, and intake below 1000 kcal/day throughout the first postoperative year [13]. The intakes recorded here lie within that envelope: 521 kcal/day at month 1 and 780 kcal/day at month 6 are both within the range recommended for the corresponding stage, and they are not described here as unusually low. What distinguishes this course is the trajectory. Intake fell from 521 to 466 kcal/day between months 1 and 3, over an interval in which the same guidance anticipates a gradual increase, and the lowest value of the series was recorded at the point at which intake should already have been rising. Energy availability therefore reached its lowest level precisely when lean tissue loss was most rapid. Restriction of this degree contributes to the loss of lean tissue when protein intake is simultaneously below target, since dietary and endogenous protein are oxidised for energy when total intake is low. Energy restriction is accordingly treated here as a contributor of at least equal standing to inadequate protein intake, without being characterised as unusually severe for this postoperative stage. The absolute protein amounts recorded (33.2–59.0 g/day) are in any case constrained by the same very small total intake, so protein inadequacy and energy inadequacy are not independent exposures in this patient. Loss of lean body mass after bariatric surgery is concentrated in the first three postoperative months and coincides with a period of reduced energy and protein intake [7]; here, the interval of most rapid fat-free mass loss was also the interval of lowest energy intake. The reliability of these estimates has to be weighed at the same time. They derive from three 24-h recalls per visit, a method subject to underreporting even when administered in person by the same dietitian throughout, and the 1541 kcal/day recorded at month 33 is difficult to reconcile with the weight regain observed over the preceding period. Underreporting at the early visits therefore cannot be excluded, and the absolute figures should be treated as approximate; what they support is the direction and the timing of the restriction rather than its exact magnitude. The patient reported no structured physical activity at any of the five assessments. Current guidance for this population recommends a combined aerobic and resistance programme, which may begin in the early postoperative period and should be continued for at least 12 to 16 weeks, and identifies the first three postoperative months as the interval in which loss of lean body mass mainly occurs and structured exercise is therefore most important [13]. Increased physical activity was advised at each postoperative visit, but the patient did not undertake a structured programme at any point, including over precisely that interval. A recent systematic review of nutritional factors in fat-free mass preservation grades the evidence linking protein intake to fat-free mass preservation as moderate in strength and notes that results are inconsistent across studies, with some finding no direct association. That review also identifies the failure of most studies to account for physical activity as a potentially significant confounder in body composition outcomes [28]. That review also identifies the first 6 postoperative months as the interval in which adequate protein intake appears most critical [28], which is the interval during which this patient’s intake was lowest. The relative contributions of inadequate protein intake, energy restriction and physical inactivity cannot be disentangled in a single patient. What this case adds is therefore not a demonstration of causation but a detailed, time-matched description of when nutritional vulnerability occurred and what it coincided with, in a patient population for which such time-matched data appear to be seldom reported.

### 4.4. Adherence to Supplementation and Long-Term Follow-Up

Regular supplementation and long-term multidisciplinary follow-up are crucial for preventing nutritional complications after bariatric surgery [7]. Sleeve gastrectomy reduces gastric volume and gastric acid secretion, and the resulting changes in intake and absorption place patients at long-term risk of deficiency in iron, vitamin B12, folate, thiamin, vitamin D and calcium, with anaemia and bone loss among the corresponding clinical consequences [7,29]. These deficiencies may develop or persist years after surgery, which is why adherence to supplementation and to lifelong biochemical monitoring determines whether they are detected and corrected [7]. The patient used supplements for only approximately one month and did not consistently attend follow-up appointments. Non-compliance with long-term follow-up has been associated with weight regain [30]. Accordingly, weight regain was observed, accompanied by self-reported increased consumption of energy-dense foods beginning approximately one year after surgery. Taken together with the earlier period of protein-rich food aversion, this case identifies two distinct, temporally separated windows of nutritional vulnerability: an early window of food intolerance, protein aversion and reduced energy intake, and a later window driven by lapsed follow-up and a shift toward energy-dense, low-protein foods, each coinciding with a measurable change in body composition or weight trajectory. Fluctuations in vitamin B12, ferritin, folate, and vitamin D concentrations during follow-up also suggest inconsistent supplement use, underscoring the importance of lifelong micronutrient monitoring in this population [7,21]. The gaps in that monitoring are themselves part of the finding rather than only a limitation of the report. A deficient 25-hydroxyvitamin D concentration of 12 ng/mL was documented at month 3 and never rechecked, so neither its correction nor its persistence over the following 30 months can be established. This is not a neutral omission: vitamin D is the only micronutrient that has been specifically studied in relation to body composition after bariatric surgery [28], although the available evidence has not demonstrated a direct association between 25-hydroxyvitamin D concentrations and body composition measures. Because vitamin D was not reassessed after month 3, its subsequent course and any potential relationship with the body composition changes described in Section 4.3 cannot be evaluated in this patient. The recommendation for lifelong micronutrient monitoring made in this report is thus supported by the present case in the negative, through what could no longer be assessed once the patient disengaged from structured follow-up. Anaemia is a related and equally persistent finding. It was present before surgery and never resolved: haemoglobin lay in the lower part of, or below, the reference range for women at all five assessments and fell below it at months 3 and 6, with low ferritin concentrations preoperatively and again at month 33, the significance of which is considered below in the light of transferrin saturation. Post-transplant anaemia is more prevalent in kidney transplant recipients than in non-transplanted patients with chronic kidney disease at the same level of glomerular filtration rate, because transplant-specific factors such as rejection episodes, infection and a proinflammatory state are superimposed on the reduced erythropoiesis that accompanies declining graft function; iron deficiency is among the contributors [31]. A meta-analysis of 85 studies found impaired allograft function to be consistently associated with post-transplant anaemia; in the same analysis, the immunosuppressive agents used in this patient were not among those associated with anaemia [29], so the regimen does not in itself account for the anaemia observed. In the present case, these coexisted with the reduced intake and altered gastrointestinal physiology that follow sleeve gastrectomy [7,21] and with the interrupted supplementation described above. Post-transplant anaemia has been associated with adverse graft and patient outcomes [32]; in this patient haemoglobin remained in the lower part of, or below, the laboratory reference range at every assessment across 33 months, fell below it at months 3 and 6, and prompted transfusion at month 3. Its relevance to the body composition findings is direct rather than incidental. In 871 kidney transplant recipients from the TransplantLines cohort, lower haemoglobin concentrations were independently associated with lower muscle mass, assessed both by 24-h urinary creatinine excretion and by BIA, with lower handgrip strength and with worse five-times sit-to-stand performance. Recipients in the lowest age- and sex-specific haemoglobin quartile had two- to sevenfold higher odds of falling into the worst quartile for these measures [33]. Anaemia in this patient should therefore not be regarded as independent of the loss of fat-free and muscle mass described in Section 4.3, nor of the absence of structured physical activity with which that loss coincided. Its position in the sequence is relevant: anaemia was present before surgery and was most severe at month 3, so it preceded and accompanied the period of greatest lean tissue loss rather than following it. Reduced oxygen-carrying capacity plausibly limited exercise tolerance and habitual activity, while the fatigue and reduced appetite that accompany anaemia may equally have limited intake. Anaemia is accordingly identified here as a potential contributor to both reduced functional capacity and lean tissue loss, and as a confounder of any apparent association between protein intake and muscle mass in this patient, rather than only as a parallel finding or a consequence. The direction of these relationships cannot be established from a single case. The ferritin trajectory requires interpretation alongside inflammatory status rather than in isolation. Ferritin is an acute-phase reactant, and its peak of 262 ng/mL at month 3 coincided with the highest recorded C-reactive protein concentration (43.6 mg/L), so inflammation is likely to have contributed to that value. The patient had also received intravenous ferric carboxymaltose 500 mg at month 2 and two units of red blood cells at month 3, both of which represent substantial iron loading shortly before that measurement. Ferritin at that visit therefore cannot be read as a measure of iron stores. The values obtained when C-reactive protein was low are more informative: ferritin concentrations of 19 ng/mL preoperatively and 26 ng/mL at 33 months were both below 30 ng/mL, a threshold used to define severe iron deficiency in kidney transplant recipients [31]. Transferrin saturation places these values in context: it was 23.5% preoperatively, at the lower limit of the range considered adequate in this population, and rose to 41.1% and 44.4% at months 3 and 6 after intravenous iron and transfusion. At month 33, however, a ferritin of 26 ng/mL was accompanied by a transferrin saturation of 34.8% and a serum iron of 87 µg/dL, so the low ferritin at that visit is not in itself evidence of absolute iron deficiency. Total iron-binding capacity was below the reference range at three of the four assessments at which it was measured; transferrin is a negative acute-phase protein, and these values, together with the fall in serum albumin over follow-up, are consistent with a contribution from inflammation and reduced protein status rather than from depletion of iron stores alone. Consistent with the limited value of ferritin in this setting, the meta-analysis cited above found no statistically significant association between ferritin concentration and post-transplant anaemia, whereas serum iron was significantly lower in anaemic recipients [29].

### 4.5. Methodological Considerations in BIA-Based Body Composition Assessment

The body composition trajectory reported here should be interpreted in the light of the hydration dependence of BIA. The non-water component of fat-free mass, calculated as fat-free mass minus total body water, was 16.9, 13.6, 12.1, 14.9 and 15.0 kg across the five assessments, while the hydration fraction of fat-free mass (total body water divided by fat-free mass) was 69.5%, 72.2%, 72.3%, 66.1% and 67.8% respectively. The lower fractions at months 6 and 33 indicate a greater deviation from the hydration assumption of approximately 73% that underlies the predictive equations used in conventional BIA [26]. The fall in the non-water component over the first three months followed by a partial rebound therefore cannot be interpreted as tissue change alone, and differences in hydration between assessments may have contributed to the observed trajectory.

This limitation is intrinsic to the technique rather than specific to this patient. Predictive equations used in conventional BIA assume a normal hydration status, and weight loss itself alters the hydration of soft tissue, a caveat acknowledged by the authors of the reference cohort used for comparison above [26]. Those authors also note that, because the bias of the method is repeatable and approximately constant when the same device and protocol are used longitudinally, serial within-patient comparisons remain informative even where absolute values are uncertain [26]. All measurements reported here were obtained on the same device by the same operator, which supports this reasoning; however, fasting state, time since last fluid intake and bladder status were not systematically standardised, and assessments were performed at different times of day.

A further consideration is specific to the present patient. Interpretation of the fluid-related outputs is particularly relevant here because renal function varied during follow-up and reduced fluid intake was documented at month 3. Extracellular and intracellular water values are therefore reported in Table 3; the ECW/TBW ratio was highest at month 6 (0.51) and lowest at 33 months (0.47), indicating variation in the relative distribution of extracellular and intracellular water across assessments. Several conditions that specifically undermine impedance-based estimation were present in this patient, and several of them clustered at the assessments on which the principal finding rests: changing graft function, a documented reduction in fluid intake at month 3, rapid weight loss throughout the early period, a shifting distribution of extracellular and intracellular water, acute systemic inflammation and an acute thrombotic event at month 3, persistent anaemia, and measurement conditions that were not standardised for fasting state, fluid intake, bladder status or time of day. BIA-derived fat-free mass cannot be equated with anatomical muscle mass under any conditions, and least of all under these. Accordingly, the 21.3% reduction reported here should be regarded as a signal requiring confirmation by a reference method rather than as a precise quantification or as a directly measured anatomical change, and the direction and timing of the changes, rather than their exact magnitude, form the basis of our interpretation.

### 4.6. Strengths and Limitations

The main strength of this case is the comprehensive 33-month follow-up with serial, time-matched assessments of body composition, dietary intake, and biochemical parameters. While fat-free mass and skeletal muscle mass loss after bariatric surgery is well documented, this case adds value by documenting when that loss occurred and which nutritional exposures—energy restriction, protein intake below target during a period of protein-rich food aversion, and absence of structured physical activity—were present over the same interval. The contribution of this report does not rest on the transplant outcome. Bariatric surgery after kidney transplantation has already been associated with lower mortality and possibly improved graft survival in a matched national cohort [11], and the favourable clinical course observed here is consistent with those data rather than novel against them. What such datasets cannot provide is what was measured in this patient: serial, time-matched body composition alongside quantified dietary protein intake. The 21.3% reduction in fat-free mass, its concentration within the first three postoperative months, and its co-occurrence with energy restriction, protein-rich food aversion and physical inactivity cannot be inferred from graft and survival endpoints, and were not among the outcomes assessed in the studies summarised in the systematic review cited above [10]. The lesson we would draw is that reassurance derived from graft and survival data does not extend to nutritional outcomes, and that body composition in this population requires monitoring in its own right. The transplant setting shaped the nutritional course of this case in several concrete ways. Aversion to protein-rich foods, which developed approximately one month after surgery and persisted through month 3, limited protein intake from food; this is a recognised problem after sleeve gastrectomy [6]. The oral protein supplement that could have closed the resulting gap, however, could not be taken regularly because the patient feared it might harm her graft, so both routes of protein delivery were closed during the same period, the second for a transplant-related reason. Setting the applicable target presented a further difficulty in this context: recommendations to increase protein intake after bariatric surgery and the attention given to renal protein load in transplant recipients have to be weighed together in the same patient, and how these two sets of recommendations should be combined after bariatric surgery in a transplant recipient does not appear to have been settled. In addition, because creatinine-based estimates of filtration may be confounded by the muscle loss documented here, the reliability of the routine method of monitoring the graft is itself uncertain over precisely the period when lean tissue was being lost most rapidly. Finally, persistent anaemia, which has been associated with lower muscle mass and lower muscle strength in kidney transplant recipients [33], coexisted with a decline in estimated filtration throughout follow-up. These features belong to the transplant setting and cannot be separated from the nutritional course described here. Muscle function was not assessed: handgrip strength, gait speed, sit-to-stand testing and the Short Physical Performance Battery were not performed, so the reduction in impedance-derived muscle mass is not corroborated by any functional measure. The biochemical panel was the routine postoperative panel of the treating centre rather than a comprehensive nutritional assessment. Serum iron, total iron-binding capacity, calcium, magnesium and zinc were measured, but not at every visit, and copper and selenium were not measured at any point, so the assessment of trace minerals was incomplete. Bone-mineral metabolism was not followed systematically and no bone density assessment was performed. Further limitations include the single-patient design, which precludes generalization, and the retrospective review of prospectively documented clinical records, which may be subject to documentation gaps. Self-reported dietary data remain susceptible to underreporting and recall bias. The reported intake of 1541 kcal/day at month 33 is difficult to reconcile with the weight regain recorded between months 6 and 33 and suggests that intake at that visit was underestimated. Body composition was assessed by BIA rather than dual-energy X-ray absorptiometry, as a DXA unit was not available at the treating institution; the implications of this for the interpretation of the present data are addressed in Section 4.5. Cystatin C was not measured at any visit and no muscle-independent estimate of filtration was available, so the effect of the muscle loss documented here on creatinine-based estimates could not be quantified in this patient. A comparable constraint applies to the immunosuppression data: tacrolimus doses, the dosing schedule, the interval between dose and sampling, the target trough range applied by the transplant unit and the mycophenolate mofetil and corticosteroid doses could not be retrieved, so the trough concentrations in Table 4 are reported without the information needed to interpret them and no conclusion about the adequacy of immunosuppression is drawn from them. A further limitation concerns the assessment of the graft itself. Allograft biopsy was not performed at any point during follow-up, and donor-specific antibodies were measured on a single occasion only, at month 4. The transient rise in serum creatinine at month 3 had resolved by month 6 without intervention, proteinuria remained below the nephrotic range, and panel reactive antibody and donor-specific antibody testing at month 4 were both negative; against this background the patient was followed by surveillance rather than by biopsy. A red cell transfusion is a recognised allosensitising exposure in transplant recipients, and the single antibody measurement available here was obtained approximately one month after the two units given at month 3; a negative result at that point cannot exclude sensitisation developing subsequently, and no further testing was performed while estimated filtration fell and proteinuria persisted. Histological contributors to the decline in estimated filtration therefore cannot be excluded, nor can antibody development after month 4, and the graft findings reported here should be read as resting on serum creatinine, estimated filtration rate, proteinuria and a single antibody assessment.

## 5. Conclusions

Although substantial weight loss was achieved after sleeve gastrectomy, early postoperative nutritional vulnerability—energy restriction, protein intake below target during a period of intolerance to protein-rich foods, poor adherence to supplementation, absence of structured physical activity and irregular nutritional follow-up—was temporally associated with marked reductions in BIA-derived fat-free mass, BIA-predicted total muscle mass and BIA-derived skeletal muscle mass, all of which reached their lowest recorded values at postoperative month 3. Fat-free mass had fallen by 21.3% from baseline at month 3 and by 20.9% at month 6, and accounted for 52.0% and 34.1% of the total weight lost at those two visits respectively; both proportions exceed the thresholds proposed for excessive fat-free mass loss [26], which are descriptive research definitions and not diagnostic criteria for muscle wasting, and the month 6 figure is numerically the more robust. These estimates are impedance-derived and hydration-dependent, and they were confirmed neither by a reference method nor by any measure of muscle function. Lean tissue loss occurred predominantly during the first three postoperative months and overlapped with, but was not strictly aligned to, the period of protein-rich food aversion and lowest protein intake; energy intake, which fell between months 1 and 3 rather than rising as the postoperative course anticipates, and the absence of structured physical activity were present over the same interval, and anaemia persisted throughout. These exposures cannot be separated from protein intake as explanations in a single case, and no causal role should be attributed to any one of them. Graft function was clinically stable but not unchanged: estimated glomerular filtration rate remained approximately 25% below the preoperative value at 33 months, and creatinine-based estimates may have overstated residual filtration in the presence of the muscle loss documented here, although this could not be verified in the absence of a muscle-independent measure of filtration. This case should therefore not be read as evidence that sleeve gastrectomy is without consequence for graft function in transplant recipients. This case underscores the potential value of adequate protein intake, structured resistance training, body composition monitoring accompanied by a simple measure of muscle function such as handgrip strength, regular vitamin-mineral supplementation, and long-term multidisciplinary nutritional follow-up in kidney transplant recipients undergoing bariatric surgery. Supervised programmes are not the only option: semi-supervised or home-based exercise, supported by education and by smart devices, is also considered acceptable [13], which may matter where attendance at structured follow-up is difficult to sustain.

### Patient Perspective

The patient reported that maintaining adequate protein intake was challenging after surgery, particularly due to intolerance to protein-rich foods such as meat, chicken, and fish, and increased taste and smell sensitivity. The patient also expressed concerns that protein supplements might adversely affect kidney function, which contributed to poor adherence to the recommended supplementation regimen.

## Figures and Tables

**Table 1:** Timeline of the patient’s clinical course.

Time	Clinical Event
2006	Diagnosed with chronic kidney disease of unknown etiology
2017	Underwent pre-emptive living-donor kidney transplantation from spouse
2017–2022	Progressive weight gain despite diet and exercise
2022	Multidisciplinary preoperative evaluation for bariatric surgery
2022	Underwent laparoscopic sleeve gastrectomy, approximately five years after transplantation
Postoperative Month 1	Nutritional assessment, body composition analysis, and biochemical evaluation
Postoperative Month 2	Intravenous ferric carboxymaltose 500 mg administered
Postoperative Month 3	Lowest recorded fat-free mass, muscle mass and skeletal muscle mass; lowest protein intake; deep vein thrombosis of the left lower limb diagnosed by Doppler ultrasonography and anticoagulant therapy initiated; two units of red blood cells transfused for symptomatic anaemia
Postoperative Month 6	Lowest recorded body weight; fat-free mass and skeletal muscle mass essentially unchanged from month 3
Postoperative Month 33	Long-term follow-up: mild weight regain, partial recovery of fat-free mass and skeletal muscle mass, no clinical signs of acute rejection

**Table 2:** Preoperative and postoperative daily energy and macronutrient intake, expressed as the mean of three 24-h dietary recalls at each visit.

	Pre-op	Post-op Month 1	Post-op Month 3	Post-op Month 6	Post-op Month 33
Energy (kcal/day)	2922	521	466	780	1541
Protein (g/day)	106.3	53.1	33.2	59.0	57.4
Protein (g/kg IBW/day)	2.00	1.00	0.63	1.11	1.08
Protein (g/kg body weight/day)	1.03	0.58	0.41	0.85	0.80
Fat (g/day)	153.6	26.0	20.4	36.2	87.9
Carbohydrate (g/day)	275.6	16.3	35.8	52.1	130.9

Abbreviations: IBW, ideal body weight, taken as the Hamwi estimate of 53.0 kg (Section 4.3) rather than the weight corresponding to a body mass index of 25 kg/m^2^ used for the weight loss metrics in Section 2.2; Pre-op, preoperative; Post-op, postoperative.

**Table 3:** Body composition parameters before and after sleeve gastrectomy.

	Pre-op	Post-op Month 1	Post-op Month 3	Post-op Month 6	Post-op Month 33
Body weight (kg)	103.4	91.8	80.7	69.4	71.9
Body mass index (kg/m^2^)	39.9	35.4	31.1	26.8	27.7
ΔBMI (kg/m^2^)	—	4.5	8.8	13.1	12.2
%TWL	—	11.2	22.0	32.9	30.5
%EWL	—	30.1	58.8	88.1	81.6
Fat-free mass (kg)	55.5	48.9	43.7	43.9	46.6
Muscle mass (kg)	52.7	46.4	41.5	41.7	44.2
Skeletal muscle mass (kg)	31.4	27.7	24.7	24.9	26.4
Body fat mass (kg)	47.9	42.9	37.0	25.5	25.3
Total body water (kg)	38.6	35.3	31.6	29.0	31.6
Intracellular water (kg)	19.2	18.2	16.1	14.2	16.6
Extracellular water (kg)	19.4	17.1	15.5	14.8	15.0
ECW/TBW ratio	0.50	0.48	0.49	0.51	0.47
Waist-to-hip ratio	1.00	1.00	0.90	0.85	0.85

All body composition values are outputs of multifrequency bioelectrical impedance analysis and are device-derived estimates rather than direct anatomical measurements; the muscle mass output represents BIA-predicted total muscle mass and is not equivalent to skeletal muscle mass measured by dual-energy X-ray absorptiometry or magnetic resonance imaging (Section 2.2 and Section 4.5). Abbreviations: BIA, bioelectrical impedance analysis; BMI, body mass index; ECW, extracellular water; %EWL, percentage excess weight loss; Post-op, postoperative; Pre-op, preoperative; TBW, total body water; %TWL, percentage total weight loss.

**Table 4:** Biochemical findings before and after sleeve gastrectomy.

	Pre-op	Post-op Month 1	Post-op Month 3	Post-op Month 6	Post-op Month 33
Creatinine (mg/dL)	1.09	1.37	1.55	1.14	1.37
eGFR (mL/min/1.73 m^2^)	63	48	42	60	47
Urine protein-to-creatinine ratio (mg/g)	356	395	325	288	423
Tacrolimus level (ng/mL)	6.5	8.3	6.3	3.3	4.8
Albumin (g/dL)	4.3	4.3	4.2	3.9	3.9
Total protein (g/dL)	6.4	6.3	6.1	5.9	6.0
Haemoglobin (g/dL)	10.8	11.3	9.4	10.3	11.1
Ferritin (ng/mL)	19	52	262	204	26
CRP (mg/L)	13.9	4.1	43.6	6.7	1.7
Vitamin B12 (pg/mL)	276	382	434	237	282
Folate (ng/mL)	4.0	6.0	5.7	7.4	NA
25-OH Vitamin D (ng/mL)	22	24	12	NA	NA
Fasting glucose (mg/dL)	91	82	103	83	86
HbA1c (%)	5.3	5.0	4.8	4.7	5.1
Urea (mg/dL)	33	30	62	37	56
Serum iron (µg/dL)	56	95	81	100	87
Total iron-binding capacity (µg/dL)	238	NA	197	225	250
Transferrin saturation (%)	23.5	NA	41.1	44.4	34.8
Calcium (mg/dL)	9.3	9.3	9.9	9.5	8.7
Magnesium (mg/dL)	1.63	1.7	1.3	1.7	1.6
Zinc (µg/dL)	104	NA	NA	82	NA

Laboratory reference ranges: haemoglobin 10.60–13.50 g/dL; ferritin 5–204 ng/mL; urine protein-to-creatinine ratio 10–107 mg/g; C-reactive protein 0–5 mg/L; serum iron 50–170 µg/dL; total iron-binding capacity 250–450 µg/dL; calcium 8.4–10.5 mg/dL; magnesium 1.6–2.6 mg/dL; zinc 60–110 µg/dL. Transferrin saturation was calculated as serum iron divided by total iron-binding capacity, multiplied by 100. The threshold of 30 ng/mL used in the text for ferritin is a clinical definition of severe iron deficiency in kidney transplant recipients rather than a laboratory limit. Several values in this table should not be read as unmodified biological measurements. Ferritin at months 3 and 6 (262 and 204 ng/mL) follows intravenous ferric carboxymaltose at month 2 and transfusion of two units of red blood cells at month 3, and the month 3 value coincides with the highest recorded C-reactive protein concentration; ferritin is an acute-phase reactant, so these values reflect iron loading and inflammation as well as iron stores. Haemoglobin at months 6 and 33 likewise follows that transfusion. Vitamin B12, folate and 25-hydroxyvitamin D were measured during a period of intermittent and incomplete supplementation. Creatinine-based eGFR is influenced by the loss of muscle mass shown in Table 3 (Section 4.1 and Section 4.5). Abbreviations: CRP, C-reactive protein; eGFR, estimated glomerular filtration rate; HbA1c, glycated haemoglobin; NA, not available; Post-op, postoperative; Pre-op, preoperative.

## Data Availability

The original contributions presented in this study are included in the article. Further inquiries can be directed to the corresponding author.

## Supplementary Materials

The following supporting information can be downloaded at https://www.mdpi.com/article/10.3390/jcm15186940/s1. CARE Checklist of Information to Include When Writing a Case Report.

## Abbreviations

The following abbreviations are used in this manuscript:

ASMBS	American Society for Metabolic and Bariatric Surgery
BIA	Bioelectrical impedance analysis
DXA	Dual-energy X-ray absorptiometry
DVT	Deep vein thrombosis
FFM	Fat-free mass
KTR	Kidney transplant recipient
MM	Muscle mass (BIA-predicted total muscle mass)
SMM	Skeletal muscle mass
