# Supplementary material for: Early Lean Tissue Loss and Nutritional Challenges Following Sleeve Gastrectomy in a Kidney Transplant Recipient: A Case Report
Source: J Clin Med. 2026 Sep 8;15(18):6940. doi: 10.3390/jcm15186940 (PMC13607079; doi:10.3390/jcm15186940)
Supplement: Supplementary file 1 [file jcm-15-06940-s001.zip › jcm-4507062-supplementary.pdf]

CARE Checklist of Information to Include When Writing a Case Report

Manuscript ID: jcm-4507062

Title: Nutritional Challenges and Changes in Body Composition Following Sleeve Gastrectomy in a Kidney Transplant Recipient: A Case Report

This case report was prepared in accordance with the CARE (CAse REport) guidelines. The table below indicates where each checklist item is reported in the revised manuscript.

No.	Topic	Item description	Reported on
1	Title	The words "case report" should appear in the title along with the phenomenon of greatest interest.	Title page
2	Key words	Two to five key words that identify topics in this case report.	Keywords, after Abstract
3a	Abstract — Introduction	What is unique and why is it important?	Abstract, Background/Objectives
3b	Abstract — Case presentation	The main symptoms of the patient; the main clinical findings; the main diagnoses and interventions; the main outcomes.	Abstract, Case Presentation
3c	Abstract — Conclusion	What are the main "take-away" lessons?	Abstract, Conclusions
4	Introduction	Brief background summary of this case referencing the relevant medical literature.	Section 1
5a	Patient information	De-identified demographic and other patient information.	Section 2.2
5b	Patient information	Main concerns and symptoms of the patient.	Sections 2.2, 3.1, 3.2
5c	Patient information	Medical, family, and psychosocial history including genetic information and relevant comorbidities, as well as relevant past interventions and their outcomes.	Sections 2.1, 2.2
5d	Patient information	Relevant past interventions and their outcomes.	Sections 2.1, 2.3
6	Clinical findings	Relevant physical examination and other clinical findings.	Sections 2.2, 3.1, 3.3
7	Timeline	Relevant data from this episode of care organized as a timeline (figure or table).	Table 1, Section 2.1
8a	Diagnostic assessment	Diagnostic methods (such as physical examination, laboratory testing, imaging, questionnaires).	Sections 2.2, 3.3, Tables 3 and 4
8b	Diagnostic assessment	Diagnostic challenges (such as access, financial, or cultural).	Sections 3.3, 4.1, 4.5, 4.6
8c	Diagnostic assessment	Diagnostic reasoning including other diagnoses considered.	Sections 4.1, 4.4
8d	Diagnostic assessment	Prognostic characteristics where applicable.	Section 4.4
9a	Therapeutic intervention	Types of intervention (such as pharmacologic, surgical, preventive, self-care).	Section 2.3
9b	Therapeutic intervention	Administration of intervention (such as dosage, strength, duration).	Section 2.3
9c	Therapeutic intervention	Changes in intervention with explanations.	Sections 2.3, 3.1
10a	Follow-up and outcomes	Clinician- and patient-assessed outcomes when appropriate.	Section 3, Tables 2–4, Figure 1
10b	Follow-up and outcomes	Important follow-up diagnostic and other test results.	Section 3.3, Table 4
10c	Follow-up and outcomes	Intervention adherence and tolerability (how was this assessed?).	Sections 3.1, 3.2, 4.2, 4.4
10d	Follow-up and outcomes	Adverse and unanticipated events.	Sections 3.1, 3.3, 4.1
11a	Discussion	Strengths and limitations in the approach to this case.	Sections 4.5, 4.6
11b	Discussion	Discussion of the relevant medical literature.	Sections 4.1–4.6
11c	Discussion	The rationale for conclusions (including assessment of possible causes).	Sections 4.1, 4.3, 4.6, 5

No.	Topic	Item description	Reported on
11d	Discussion	The primary "take-away" lessons from this case report.	Sections 4.6, 5
12	Patient perspective	The patient should share their perspective on the treatments they received.	Patient Perspective section
13	Informed consent	Did the patient give informed consent? Provide if requested.	Informed Consent Statement

Reference: Gagnier JJ, Kienle G, Altman DG, Moher D, Sox H, Riley D; CARE Group. The CARE guidelines: consensus-based clinical case report guideline development. J Clin Epidemiol. 2014;67:46-51.
